# Electrically Controlled Self-Focusing and Self-Localization in the Guided Channels

**DOI:** 10.3390/polym11111767

**Published:** 2019-10-27

**Authors:** Bing-Yau Huang, Yi-Hsiu Wu, Shuan-Yu Huang, Chie-Tong Kuo

**Affiliations:** 1 Department of Physics, National Sun Yat-sen University, Kaohsiung 804, Taiwan; flyfishss31@gmail.com (B.-Y.H.); yihsiu0315@gmail.com (Y.-H.W.); 2 Department of Optometry, Chung Shan Medical University, Taichung 402, Taiwan; 3 Department of Ophthalmology, Chung Shan Medical University Hospital, Taichung 402, Taiwan; 4 Department of Optometry, Shu-Zen Junior College of Medicine and Management, Kaohsiung 821, Taiwan; 5 Innovation Incubation Center, Shu-Zen Junior College of Medicine and Management, Kaohsiung 821, Taiwan

**Keywords:** liquid crystals (LCs), guided channels, beam coupling, electrically controllable, self-focusing

## Abstract

In this paper, we demonstrate the potential of liquid crystals (LCs) on the applications of small, simple, and tunable optical guided channels. Experimental results show that three operation modes of beam coupling can be achieved, depending on the feature of the electrically controllable refractive index, the incident position, and the specific design of electrodes. The dependence of the beam polarization on self-focusing and coupling effect are also discussed. The electrically controllable self-focusing and beam coupling are highly potential on integrated photonic circuits.

## 1. Introduction

Manipulation of light has been an important topic since there is a vast range of potential applications of light on various fields, such as displays and holography, imaging and movements of micro/nano objects, data storages, data communications, and integrated photonic circuits [1,2,3,4,5,6,7,8,9,10,11,12]. All of them have played significant roles in human communities. For data communications, guidance or transmission of light is carried out using optical fibers. The mechanism of guiding light in optical fibers is the total reflection of light via the difference of refractive indices (Δ*n*) between the core and cladding of the optical fiber [13]. However, optical fibers are not suitable to be embedded into photonic circuits and lack tunability, which also limits their application for photonics computing. 

Liquid crystal (LC) is an interesting material with a tunable refractive index as well as birefringence, and has been extensively applied on displays and other photonic devices [14,15,16,17,18]. A LC with positive dielectric anisotropy (Δ*ε* > 0) will reorient to the direction of the external electric field and thus the refractive index will vary [19]. The electrically controllable refractive index of LCs has been employed to develop a variety of optical devices, such as lens, waveplates, and gratings [20,21,22,23,24,25]. In addition, the LC-based optical devices have the advantageous feature of miniature size, which is highly suitable to be integrated into photonic circuits [26,27,28]. The tunable optical features of LCs have also been adopted for the developments of waveguides [29,30,31], beam steering [32,33,34], and beam coupling [35,36,37].

In this paper, we demonstrate the potential of LCs on the applications of small, simple, and tunable optical guided channels. Experimental results show that three operation modes of beam coupling can be achieved, depending on the feature of the electrically controllable refractive index, the incident position, and the specific design of electrodes. The dependence of the beam polarization on self-focusing and coupling effect are also discussed. The electrically controllable self-focusing and beam coupling have high potential for integrated photonic circuits.

## 2. Materials and Methods

The platforms for demonstrating self-focusing and self-localization in guided channels in this work were fabricated by injecting nematic liquid crystals E7 (from Merck, Darmstadt, Germany) into empty cells with grating-like electrode channels. The liquid crystal E7 is a mixture including four compounds, pentylcyanobiphenol (5CB), heptylcyanobiphenol (7CB), 4-cyano-4′-n-octyloxy-1,1′-biphenyl (8OCB), and 4-cyano-4′′-n-pentyl-1,1′,1′′-terphenyl (5CT), with the proportions of 51:25:16:8 in weight [38]. The temperature range of E7 at nematic phase is about −10–60 °C. The liquid crystal E7 was employed for the experiment as received, without further purification. To fabricate the empty cell, one glass substrate with grating-like electrode channel and one ITO-coated glass substrate were assembled with two 38-μm-thick Mylar spacers. The substrates were coated with alignment layers (SE-130, from Nissan, Tokyo, Japan) and anti-parallel rubbed. The width and space of the grating-like electrode channels were 15 and 30 μm, respectively.

**Figure 1:**
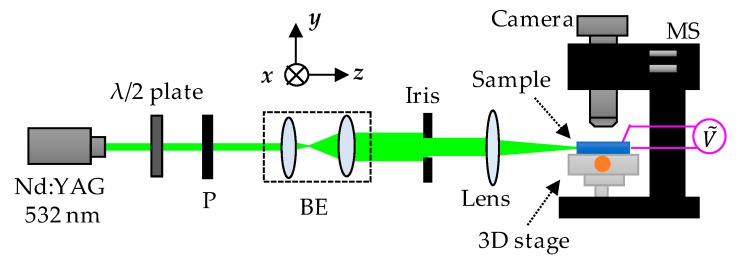
Schematic illustration of the experimental setup. P: polarizer, BE: beam expender, and MS: microscope.

A Nd:YAG laser with wavelength of 532 nm was used as the probe beam. The laser beam passed through a λ/2 plate, a polarizer (P), a beam expender (BE), an iris, and a focusing lens to feed into the side of the sample, as shown in Figure 1. An electrically controllable three-axis translation stage (3D stage) was used to precisely adjust the position of the sample, and thus the laser beam could be correctly introduced into the channel. A 1 kHz alternating current (AC) electric field of square wave was applied on the sample to induce the reorientations of liquid crystal molecules and thus to form the guided channels. The propagated beam was observed by a color camera (EOS 700D, Canon) through a microscope (MS). The captured images were analyzed to extract the intensity distribution of the coupled beam in each channel.

## 3. Results and Discussion

**Figure 2:**
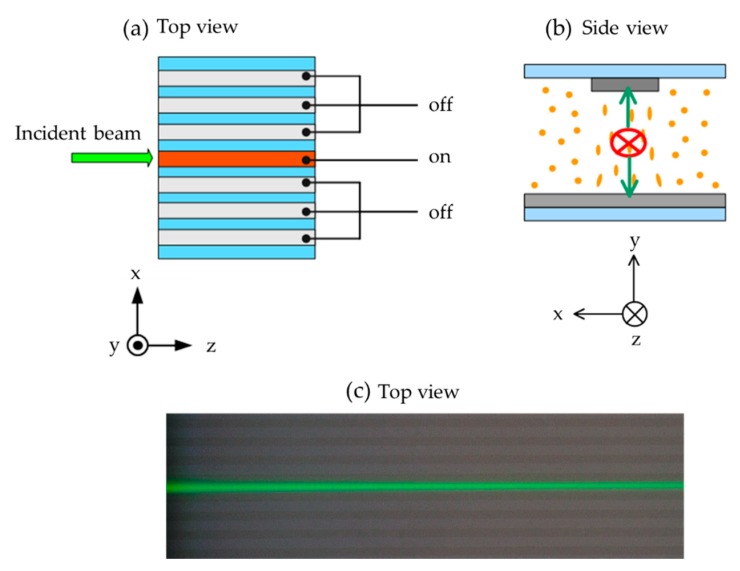
The incident beam in a single channel, (**a**) top view and (**b**) back view of the schematics for the incident beam and the sample schematic. (**c**) The top view image captured by camera. The light gray stripes are the electrode areas of the sample.

Figure 2a,b schematically presents the incident position of the probe beam from top view (along y-axis) and the reorientations of LCs under applied voltage observed from the incident direction (along z-axis), respectively. First, the voltage is only applied to the central channel and the laser beam is introduced from the left side of the sample, as shown in Figure 2a. The corresponding observed image is shown in Figure 2c. The polarization of the laser beam is fixed to be parallel to the y-axis (s-polarization). As the voltage gradually increases, the directors of LCs in the channel can be reoriented to be parallel to y-axis, as shown in Figure 2b. Therefore, the incident light experiences the larger refractive index in the channel with the existence of electric field.

**Figure 3:**
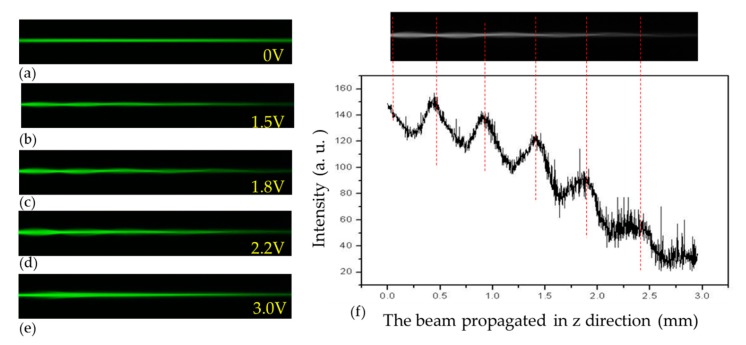
The evolution of the self-focusing in a single channel with the applied voltages of (**a**) 0, (**b**) 1.5, (**c**) 1.8, (**d**) 2.2, and (**e**) 3 V. (**f**) The propagation of laser beam in a single channel with the applied voltage of 1.8 V and the corresponding intensity distribution.

Figure 3 presents the evolution of the self-focusing phenomenon in a single channel with the applied voltage from 0 to 3 V. When the voltage is 0 V, the director of the LC molecules is parallel to the z-axis (Figure 3a). When the voltage applied is 1.5 V, the LC molecules are only slightly reoriented by the electric field and the self-focusing phenomenon is not obvious (Figure 3b). As the voltage is increased to 1.8 V, the electric field drives the LC molecules in the channel and results in a gradient of the refractive index around the channel and thus the self-focusing phenomenon of the propagated beam through the channel, as shown in Figure 3c. As the voltage is increased to 3 V, the LC molecules in the channel are almost reoriented to the direction of the y-axis by the electric field. However, the electric field is also large enough to reorient the LC molecules outside the channel and leads to the decreased difference of refractive index around the channel boundary. The decreased difference in refractive index (Δ*n)* perceived by the polarized beam gradually diminishes the self-focus phenomenon, as shown in Figure 3e. Figure 3f presents the propagation of laser beam in a single channel with the applied voltage of 1.8 V. The laser beam is periodically converged and then diverged in the propagation process, and the distance between the nodes is about 0.48 mm. Due to the difference in the distribution of the refractive index of the medium, the effect of light leakage or scattering reduces the intensity of laser beam gradually during the propagation along the z-axis.

**Figure 4:**
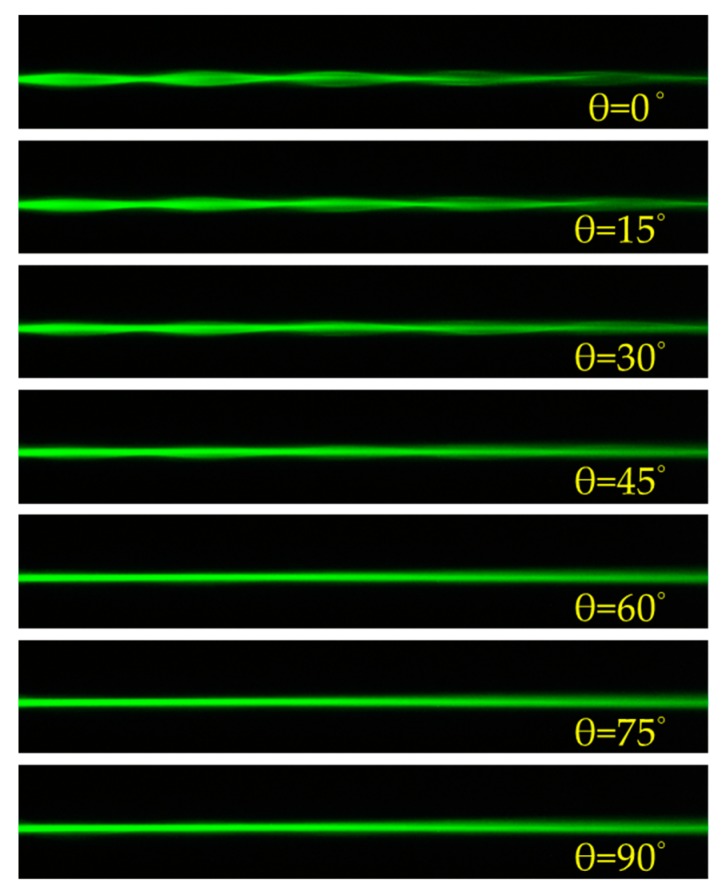
The polarization dependence of the incident laser beam on the self-focusing with the applied voltage of 1.8 V.

Figure 4 presents the polarization dependence of the incident laser beam on the self-focusing phenomenon with the applied voltage of 1.8 V. The polarization of the incident beam is rotated from 0° (y-axis) to 90° (x-axis) by a step of 15°. Here, we define θ as the angle between the direction of the polarized beam and the director of the LC molecules. When θ is 0°, the laser beam experiences the largest difference of the refractive index (Δ*n*) around the channel, and thus the self-focusing effect is obvious. As the angle θ gradually increases, the difference of the refractive index (Δ*n*) gradually decays and results in the reduction of the self-focusing effect. When θ is increased to 90°, the difference of the refractive index almost approaches to zero around the channel, and thus no self-focusing effect can be observed.

**Figure 5:**
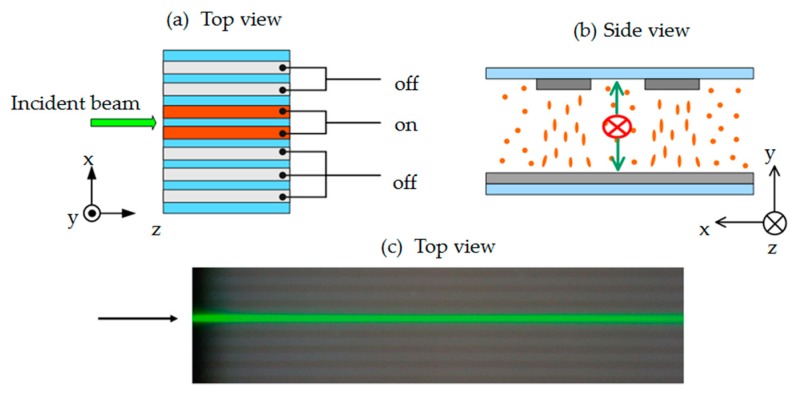
The incident beam is introduced into a non-electrode channel, (**a**) top view and (**b**) back view of the schematics for the incident beam and the sample schematic. (**c**) The top view image captured by camera.

Now we discuss the case in which the incident beam is introduced into the non-electrode area, and the voltage is applied to the two adjacent channels, as shown in Figure 5a. Figure 5b schematically illustrates the orientations of the LC molecules in the sample from the back view. Figure 5c is the top view image captured by camera (without applied voltage). The polarization of the incident laser beam is parallel to the y-axis. As the voltage gradually increases, the director of LC molecules in the two channels can be reoriented to be parallel to y-axis, as shown in Figure 5b, resulting in two waveguides in the electrode areas. Therefore, the energy of the incident light is expected to be coupled into the two electrode channels with applied voltage.

**Figure 6:**
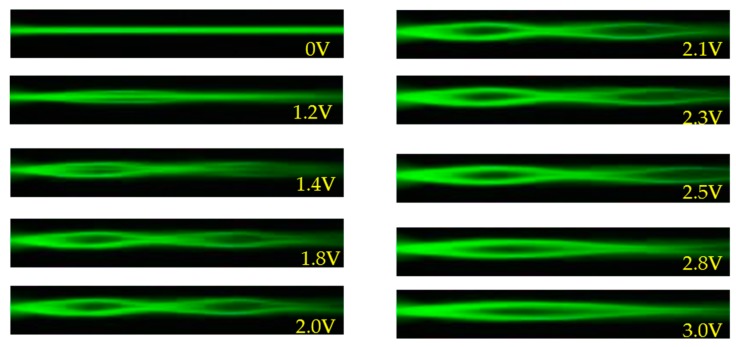
The intensity distributions of the incident beam introduced into the non-electrode area with the applied voltage from 0 to 3 V.

Figure 6 presents the intensity distributions of the incident beam with the applied voltage from 0 to 3 V. When the applied voltage is 1.2 V, the LC molecules are reoriented by the applied electric field, and part of the incident light is initially coupled into the electrode channels. When the applied voltage is higher than 1.8 V, the coupling effect becomes obviously. It can be speculated waveguide-like structures are formed between the electrode and non-electrode regions. As the applied voltage gradually increases, the laser beam experiences the larger refractive index in the electrode regions than that in the original channel (non-electrode channel). The larger refractive index in the electrode channels indicates that the velocity of light in the electrode channels will become slower. The differences of refractive indices and velocity of light in the electrode and non-electrode regions results in the change of the wavefront of the propagated beam. The change of the wavefront leads to the deflection of light, which in turn couples the propagated light into the electrode channels and confines the light in the channels when the applied voltage from 1.8 V to 2.5 V. However, the light coupling and waveguiding effects become much weaker when the applied voltage is further increased. This phenomenon is due to the fact that some LC molecules in the non-electrode region are also reoriented to the y-axis, resulting in the difference of refractive index among two areas decreases.

**Figure 7:**
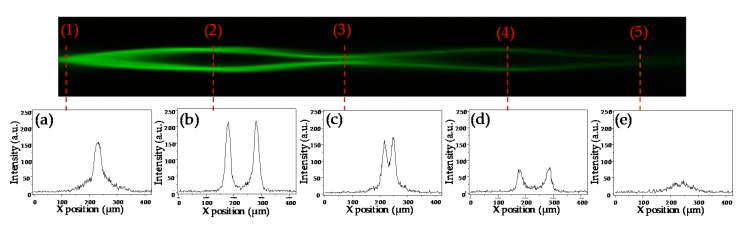
Captured image of the laser beam and corresponding intensity distributions at (**a**) z = 0 mm, (**b**) z = 0.8 mm, (**c**) z = 1.5 mm, (**d**) z = 2.3 mm, and (**e**) z = 2.8 mm, respectively, with the applied voltage of 2.1 V.

Figure 7 shows that the laser beam is coupled and confined in two electrode channels with the applied voltage of 2.1 V. The intensity distributions of the laser beam at five marked positions of the laser beam along the propagation direction (z-axis) are analyzed. At z = 0 mm (position (1)), the energy of the laser beam is concentrated in the original channel; at z = 0.8 mm (position (2)), z = 1.5 mm (position (3)), and at z = 2.3 mm (position (4)), the deflection of the laser beam to the electrode channels can be clearly observed. At z = 2.8 mm (position (5)), the intensity is very low. The gradually decreased intensity of the laser beam is due to the energy loss during propagation, which could be caused by scattering.

**Figure 8:**
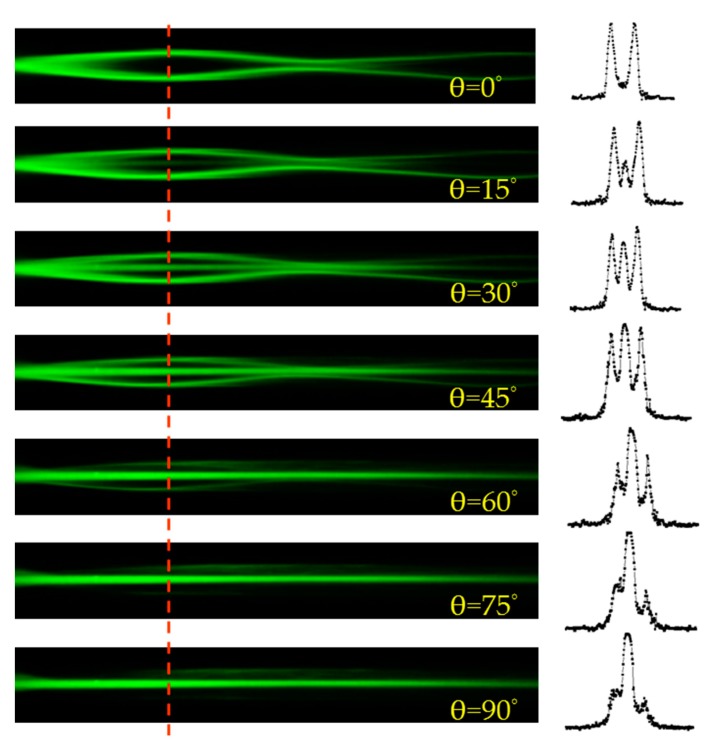
The polarization dependence of laser beam coupling into electrode channels with the applied voltage of 2.1 V. The intensity distributions of the laser beam at the position of z = 0.8 mm is also displayed.

The polarization dependence of laser beam coupling with the applied voltage of 2.1 V and the corresponding intensity distributions of the laser beam at the position of z = 0.8 mm are presented in Figure 8. When θ is 0°, the laser beam experiences the largest difference of the refractive index (Δ*n*) between the electrode and the non-electrode channels, and thus the energy of the laser beam can be coupled into the electrode channels. As the angle θ gradually increased from 15° to 75°, the difference of the refractive index (Δ*n*) gradually decreases. Therefore, the intensity of the laser beam in the original channel gradually increases, while the intensity of the laser beam coupled into the electrode channels gradually reduces. When θ is 90°, the Δ*n* experienced by the laser beam approaches zero; as a result, the propagation of the laser beam is hardly observed in the electrode channels.

**Figure 9:**
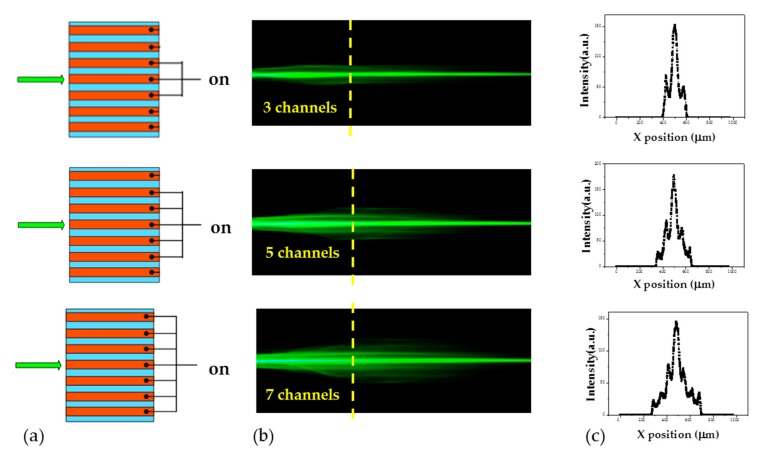
Beam coupling into multiple channels. (**a**) Schematics for the incident position of the laser beam and the electrode channels with the applied voltage of 2.8 V. (**b**) The top view of the propagation of laser beams coupled into three, five, and seven channels, respectively. (**c**) The intensity distributions of the laser beam at the positions labeled by the yellow dash lines shown in (**b**).

The previous results indicate that the incident beam can be confined and self-focused in a single channel or coupled into two channels by electric field, depending on the incident position. At specific conditions, the incident beam can be coupled into multiple channels. Figure 9 presents the cases for when the incident beam is introduced in the central electrode channel, and simultaneously the voltage of 2.8 V is applied to three, five, and seven electrode channels, respectively. As shown in Figure 9b,c, the laser beam can be coupled into the electrode channels by adjusting the voltage to each channel.

## 4. Conclusions

This paper demonstrates the electrically controllable self-focusing and self-localization phenomena in guided channels of a specific LC cell with designed electrodes. The self-focusing phenomenon is optimal with the applied voltage of 1.8 V, and disappears when the electric field is large enough to drive the LC molecules around the boundaries of the electrode channels. The polarization of the laser beam has a significant effect on the self-focusing phenomenon. When the incident beam is introduced into a non-electrode channel, and the voltage is applied to the two adjacent channels, the energy of the incident beam can be coupled into the two electrode channels. The polarization dependent coupling of the laser beam can be used to regulate the distribution of the light intensities in the electrode channels. Simultaneous beam coupling into multiple channels is also demonstrated by introducing the laser beam into the central electrode channel and applying voltage of 2.8 V to multi-electrode channels. The results reported herein provide a new insight into the development of electrically-controllable guided channels in LCs.
